# Onset risk factors for youth involvement in cyberbullying and cybervictimization: A longitudinal study

**DOI:** 10.3389/fpsyg.2022.1090047

**Published:** 2023-01-19

**Authors:** Anna Sorrentino, Alessia Esposito, Debora Acunzo, Margherita Santamato, Antonio Aquino

**Affiliations:** ^1^Department of Psychology, University of Campania Luigi Vanvitelli, Caserta, Italy; ^2^Department of Neuroscience, Imaging and Clinical Sciences, Chieti-Pescara University, Chieti, Italy

**Keywords:** cybervictimization, cyberbullying, risk factors, onset, aggressive behaviors

## Abstract

**Introduction:**

Cyberbullying and cybervictimization are spread worldwide, and due to COVID-19, an increasing number of children and adolescents have been impacted. Since the beginning of the twenty-first century, research has investigated and highlighted the key risk factors for cyberbullying and cybervictimization, and numerous anti-cyberbullying prevention and intervention programs have been developed and assessed for their efficacy. Despite this, no studies have specifically focused on the individual, relational, and contextual risk factors associated with the onset of youth involvement in cyberbullying and cybervictimization.

**Methods:**

To address this lacuna, 333 Italian students aged 10–16 years (M = 12.16, SD = 1.35) were involved in a year-long longitudinal study and filled in the anonymous online actuarial Tabby Improved Checklist two times with a 6-month interval. Onset risk factors for cyberbullying and cybervictimization have been separately analyzed by excluding all students involved in cyberbullying from the original sample or in the cybervictimization baseline (T1).

**Results:**

The results showed that being male, being involved in school bullying, having low levels of awareness of online risk, and having high levels of affective empathy were all significant onset risk factors for cyberbullying. Similarly, being male, being involved in school bullying and victimization, having high levels of affective empathy, and moral disengagement were onset risk factors for cybervictimization.

**Conclusion:**

Given the negative psychological and behavioral consequences of cyberbullying and cybervictimization, this article includes discussions on practical and policy implications for future research, stressing the need to develop, implement, and evaluate the effectiveness of primary prevention programs addressing and managing onset risk factors for cyberbullying and cybervictimization.

## Introduction

Cyberbullying could be defined as “an aggressive, intentional act carried out by a group or individual, using electronic forms of contact, repeatedly and over time against a victim who cannot easily defend him or herself” (Smith et al., 2008, p. 376). Within a short time, it has become a socially worrying phenomenon, spreading rapidly and in tandem with the adoption of new technologies and smartphones among young people and teenagers. Technological innovation expanded school bullying into cyberspace (Lee et al., 2018). The involvement of youngsters and children in cyberbullying and cybervictimization has become a matter of global attention and concern, with cybervictimization rates increasing from 13.9 to 57.5% and cyberbullying up from 6.0 to 46.3%, indicating, as underlined by Zhu et al. (2021), a significant increase of prevalence rates of such behaviors among youth in the last 5-year period.

In Europe, the EU KIDS 2020 report highlighted that cybervictimization prevalence rates ranged between 7.0 and 40.0%, with Slovakian adolescents reporting a lower involvement in cybervictimization. On average, cybervictimization prevalence across Europe is 20.0%, with Poland youngsters reporting the highest experience of cybervictimization at 40.0%.

Similar results were found concerning cyberbullying involvement, with prevalence rates across European countries ranging between 10.0 and 20.0%, with the highest involvement in cyberbullying reported by Polish adolescents (38.0%) (Smahel et al., 2020).

The involvement of youth and children in cyberbullying and cybervictimization increased during the COVID-19 pandemic due to the lockdown and the increased time spent at home using electronic devices. Lobe et al. (2021) analyzed the changes in cyberbullying trends by comparing the pre-pandemic period with the pandemic period, surveying 1,028 Italian students aged between 10 and 18 years. The results showed that youth involvement in cyberbullying and cybervictimization during the pandemic increased by 49.0 and 50.0%, respectively. Furthermore, results showed that 28.0 and 41.0% of participants reported being involved at least once in cybervictimization and cyberbullying, respectively. Similar results were found by other studies and researchers, confirming the role of the pandemic in increasing the risk of involvement in cyberbullying and cybervictimization among youth (Mkhize and Gopal, 2021; Shin and Choi, 2021; Utemissova et al., 2021; Trompeter et al., 2022). Worldwide, the increasing involvement of children and adolescents in such aggressive and deviant behaviors as perpetrators and victims stresses the importance of prevention and intervention strategies, as cyberbullying should be considered a public health problem. Several studies have highlighted the numerous long-term negative consequences associated with involvement in cyberbullying and cybervictimization (Camerini et al., 2020).

Psychological and mental problems such as depression, anxiety, and low levels of self-esteem (Kowalski et al., 2014; Eyuboglu et al., 2021) and life satisfaction (Kowalski et al., 2014), psychosocial difficulties, and self-injurious behaviors (Eyuboglu et al., 2021) are among the most reported psychological and mental outcomes associated with youth involvement in cyberbullying. Moreover, as underlined by the results of a recent systematic review by John et al. (2018), students involved in cyberbullying are at a greater risk of suicidal ideation and attempts of suicide than students who are not involved. Significantly, substance abuse such as alcohol, tobacco, and cannabis smoking was among the main behavioral negative consequences associated with youth involved as cyberbullies (Kowalski et al., 2014; Yoon et al., 2019; Eyuboglu et al., 2021; Pichel et al., 2022).

The Cambridge Study in Delinquent Development (CSDD) showed that being involved in school bullying at the age of 14 predicted violent convictions between ages 15 and 20, low job status at the age of 18, drug use at the age of 27–32, and an unsuccessful life at age 48 (Farrington and Ttofi, 2011). But as far as we know, to date, there are no studies that have assessed the longitudinal impact of involvement in cyberbullying behaviors despite such behaviors being strongly associated with attitudes favorable to the transgression of social norms (Romero-Abrio et al., 2019) and a greater risk of dating deviant and violent peers (Kim et al., 2017).

Cybervictims, on the other hand, have similarly reported several self-rated poor mental health (Sampasa-Kanyinga et al., 2020) and psychological symptoms (Yang et al., 2021); examples of negative psychological consequences include depression (Eyuboglu et al., 2021; Hu et al., 2021; Tran et al., 2021), post-traumatic stress symptoms (Baldry et al., 2019), anxiety, and psychosocial difficulties (Eyuboglu et al., 2021). John et al. (2018), in their systematic review of 33 studies, reported that cybervictims are, respectively, 2.35, 2.15, and 2.57 times more at risk when compared with non-cybervictims of self-injurious behaviors, suicidal ideation, and attempts of suicide. More recent studies also reported similar results (Sampasa-Kanyinga et al., 2020; Eyuboglu et al., 2021; Yang et al., 2021; Buelga et al., 2022). Substance abuse (such as alcohol, tobacco, and cannabis smoking) (McCuddy and Esbensen, 2017; Graham and Wood, 2019; Sampasa-Kanyinga et al., 2020; Pichel et al., 2022), sex with multiple partners (Graham and Wood, 2019), low school achievements (Guo, 2016), and delinquency (Nasaescu et al., 2020) are among the major behavioral negative consequences associated with the experience of cybervictimization.

Due to the increasing trend in cyberbullying and cybervictimization and considering the several negative psychological and behavioral consequences associated with it, recent research is exploring the possible influence of multiple individual, relational, and contextual risk factors associated with cyberbullying and cybervictimization (Hellsten et al., 2021).

Concerning the role of gender, most studies found that being male to be an individual risk factor for cyberbullying (Baldry et al., 2015; Guo, 2016; Barlett et al., 2021; Giordano et al., 2021), while few other studies highlighted that girls were more involved in cyberbullying than boys (Kowalski and Limber, 2007; Li, 2007; Vandebosch and Van Cleemput, 2009; Låftman et al., 2013), and being female was found to be a significant risk factor for cybervictimization (Pettalia et al., 2013; Morin et al., 2018; Alhajji et al., 2019; Kim et al., 2019; Smith et al., 2019; Aizenkot and Kashy-Rosenbaum, 2021; Eyuboglu et al., 2021). While Connell et al. (2014) found that girls were more involved than boys in cyberbullying and cybervictimization; other studies found that boys showed greater involvement in cyberbullying and cybervictimization than girls (Huang et al., 2019; Rao et al., 2019). However, few studies found no significant gender difference in both cyberbullying and cybervictimization (Park et al., 2014; Chang et al., 2015a; Sanmartín Feijóo et al., 2021), and few others did not find gender differences in cybervictimization (Sorrentino et al., 2019).

Several studies highlighted that high levels of moral disengagement were a significant individual risk factor for youth involved in cyberbullying (Bauman, 2010; Pozzoli et al., 2012; Guo, 2016; Yang et al., 2018; Bartolo et al., 2019; Wang et al., 2019), while other studies found that involvement in both cyberbullying and cybervictimization was associated with high levels of moral disengagement (Pornari and Wood, 2010; Kowalski et al., 2014; Chen et al., 2017; Parlangeli et al., 2020) with cybervictims reporting higher levels of hostile attributional bias and cyberbullying scoring higher in moral justification (Pornari and Wood, 2010).

A few studies that investigated the role of the incorrect use of the internet, low levels of awareness of risky online behaviors, and online security procedures among youth involved in cyberbullying and cybervictimization found a significant correlation resulting in increasing the risk of children and youth being involved both as cyberbullies and cybervictims (Fanti et al., 2012; Chang et al., 2015a; Camerini et al., 2020; Craig et al., 2020).

Regarding empathy as an individual risk factor for cyberbullying and cybervictimization, contrasting results emerged. The majority of studies found a positive association between low levels of empathy and involvement in cyberbullying (Steffgen et al., 2011; Topcu and Erdur-Baker, 2012; Casas et al., 2013; Kowalski et al., 2014; Baldry et al., 2015; Zych et al., 2019b; Sorrentino et al., 2021). In particular, some studies highlighted that both low levels of affective and cognitive empathy were significant risk factors for children and adolescents' involvement in cyberbullying (Ang and Goh, 2010; Del Rey et al., 2016; Zych et al., 2019b). On the contrary, few other studies did not find any significant association between both affective and cognitive empathy and cyberbullying (Graf et al., 2019).

Few studies examined the relationship between levels of empathy and cybervictimization; some of them found no significant associations (Steffgen and König, 2009; Kowalski et al., 2014), while other studies found low empathy to be a significant risk factor for cybervictimization among youth (Schultze-Krumbholz and Scheithauer, 2009). A recent systematic review and meta-analysis of 25 studies (Zych et al., 2019b) found no significant association between levels of empathy and cybervictimization, but when affective and cognitive empathy were considered separately, cybervictims scored higher compared to non-cybervictims on affective empathy while no significant association was found between cognitive empathy and cybervictimization.

One of the primary triggers for cyberbullying and cybervictimization is involvement in school bullying (Ansary, 2020; Estévez et al., 2020; Vismara et al., 2022). Studies investigating the relationship between adolescents' role in school bullying and victimization and cyberbullying and cybervictimization lead to contradicting observations, with the majority of them hypothesizing a substantial overlap and role continuity between the two types of peer aggression (Raskauskas and Stoltz, 2007; Del Rey et al., 2012; Fanti et al., 2012; Hemphill et al., 2012, 2015; Low and Espelage, 2013; Sticca et al., 2013; Hemphill and Heerde, 2014; Kowalski et al., 2014, 2019; Baldry et al., 2015; Waasdorp and Bradshaw, 2015; Athanasiades et al., 2016; Festl, 2016; Guo, 2016; Chen et al., 2017; Lazuras et al., 2017; Wolke et al., 2017; Jiménez, 2019; Leemis et al., 2019; Wang et al., 2019; Cosma et al., 2020; Khong et al., 2020; Oriol et al., 2021; Pichel et al., 2021; Rodríguez-Álvarez et al., 2021; Chudal et al., 2022). On the contrary, other studies found that school victims were more likely to be involved in cyberbullying (Ybarra and Mitchell, 2004; Kowalski et al., 2012; Cuadrado-Gordillo and Fernández-Antelo, 2014, 2019; Baldry et al., 2016; You and Lim, 2016; Lazuras et al., 2017).

Despite several individual risk factors, studies also underlined the importance of crucial parental, peer, and contextual protective factors for the involvement of youth in cyberbullying and cybervictimization (Zych et al., 2019a).

Regarding parental protective factors, contrasting results have emerged on parents' involvement in giving clear rules and monitoring their children's online life (López-Castro and Priegue, 2019; Zhu et al., 2021), with the majority of them emphasizing the protective role of such parental mediation strategy in preventing cyberbullying and cybervictimization (Hemphill and Heerde, 2014; Kowalski et al., 2014; Chang et al., 2015b; Khurana et al., 2015; Hong et al., 2016; Doty et al., 2018; Zych et al., 2019a). Few studies investigated the role of parental support in preventing and reducing both cyberbullying and cybervictimization (López-Castro and Priegue, 2019; Zych et al., 2019a; Camerini et al., 2020) as underlined in a recent study involving 774 Turkish students carried out by Ates et al. (2018) which found that parental support was a significant protective factor both for the involvement in cyberbullying and cybervictimization. Other studies found a different pattern between cyberbullies and cybervictims, highlighting that high levels of parental support served as protective factors only for cybervictims (Doty et al., 2017; Canestrari et al., 2021; Arató et al., 2022).

A recent meta-analysis by Zych et al. (2019a) showed that feeling supported by peers could be a protective factor against involvement in cyberbullying and cybervictimization. Similar results were also reported by Ates et al. (2018) and Arató et al. (2022), while according to Guo et al. (2021), high levels of peer support at school was a protective factor only for cybervictimization.

At the contextual level, several studies focused on the role that perceived school climate could have in affecting or being associated with both cyberbullying and cybervictimization, with the majority of the existing research indicating that a perception of a positive and safe school climate was associated with a decreased risk of being involved both as cyberbullies and cybervictims (Guo, 2016; Zych et al., 2019a; Camerini et al., 2020; Zhu et al., 2021).

Despite several studies investigating the risk and protective factors for cyberbullying and cybervictimization, the majority were cross-sectional, with only 76 studies adopting a longitudinal design (Camerini et al., 2020).

As underlined by Polanin et al. (2021) in their systematic review and meta-analysis of 50 studies concerning the effectiveness of cyberbullying preventive programs in reducing cyberbullying and cybervictimization, none of them included the concept of antisocial onset in preventing youth involvement in cyberbullying and cybervictimization. These results emphasize the need to investigate and include onset risk factors for involvement in cyberbullying and cybervictimization to develop and implement preventive anti-cyberbullying programs and evaluate their effectiveness in reducing cyberbullying and cybervictimization over time (Lan et al., 2022).

To analyze the onset of cyberbullying and cybervictimization behaviors, we adopted the same theoretical framework as Baldry et al. (2015). Combining the ecological system theory (Bronfenbrenner, 1977, 1979) and the threat assessment approach (Fein et al., 1995; Borum et al., 1999), allows the identification of significant risk factors for cyberbullying and cybervictimization by collocating them in their respective ecological system, and investigating how they operate and interact with each other, influencing the onset of cyberbullying and cybervictimization behaviors.

Bearing this in mind, the present study aims to investigate how individual, parental, peer, and school risk factors affect the onset of youth involvement in cyberbullying and cybervictimization by conducting a short-term longitudinal study.

In line with the international literature, we expected that significant risk factors for the onset of cyberbullying were being male, having low levels of awareness of online risky behaviors and both cognitive and affective empathy, high levels of moral disengagement, being a school bully, feeling not supported by parents and monitored about their online activities, perceiving low levels of support by peers, and a negative school climate.

Concerning risk factors for cybervictimization, we expected that cybervictims were more likely to be female, with low levels of awareness of online risky behaviors, high levels of both cognitive and affective empathy, low levels of moral disengagement, being victims of school bullying, feeling not supported by parents and monitored about their online activities, perceiving low levels of support by peers, and a negative school climate.

## Materials and methods

### Participants

The initial sample consisted of 455 students randomly recruited from five schools participating in a short-term longitudinal study.

Eventually, 333 students were included in the analyses as they had taken part and completed phases T1 and T2 (73% of the initial sample), and their questionnaire could be correctly matched. Attrition analysis with the dropped-out samples showed significant differences with regard to school victimization, *F*_(1, 453)_ = 14.809, *p* < 0.001 (the drop-out sample *M* = 1.25, *SD* = 2.85; the final sample *M* = 2.63, *SD* = 3.57) and perceived parental support, *F*_(1, 452)_ = 5.05, *p* = 0.025 (the drop-out sample *M* = 6.46, *SD* = 3.32; the final sample *M* = 7.44, *SD* = 4.36). No significant differences were found concerning involvement in school bullying, levels of moral disengagement, cognitive and affective empathy, awareness of online risks, perceived peer support, parental online monitoring strategies, and school climate. The dropping out of 122 students was due to mistakes in filling in the matching ID code that students had to create to guarantee their anonymity or absence on the day of data collection.

Of all students, 47.7% were male and 52.3% female, and aged between 10 and 16 years old (*M* = 12.27, *SD* = 1.42).

Regarding the use of cyber communication, 94.5% of all students reported at least one profile on a social network. Of those with a profile, 4.4% personally knew only a few of their online contacts, and 63.5% of students, on average, spent 1–4 h a day online. Concerning students' experiences of cyberbullying and cybervictimization at T1, 11.0% reported cyberbullying others at least once in the past 6 months, and 36.0% have been cybervictimized at least once in the past 6 months.

### Measures

The online Tabby Improved Checklist was developed by analyzing the results of a review of the international literature on risk factors for youngsters' involvement in cyberbullying and cybervictimization and how these risk factors operate and interact at different levels according to the ecological theoretical framework. For the short-term predictive ability of the risk, the previous instrument (Baldry et al., 2018; Sorrentino et al., 2018) was used for the present study.

The Tabby Improved Checklist consists of 12 scales and 130 items, measuring ontogenetic, microsystem, and community-level risk factors. For the purpose of the present paper, the following scales and items were analyzed.

Participants' involvement in cyberbullying and cybervictimization was measured by adopting the taxonomy by Willard (2007): flaming, denigration, impersonation, outing, and exclusion (five items for cyberbullying and five items for cybervictimization for each scale). Students rated their experiences of cyberbullying and cybervictimization on five-point Likert scales ranging from 0 = “it has never happened in this period” to 4 = “it happened several times a week.” Example items: “I pretended to be someone else, created a fake profile in order to send or post damaging messages about another person,” “I disclosed online private information or images without the person's consent,” and “I was actively engaged in excluding someone from an online group.” To measure the onset of cyberbullying and cybervictimization, scores on the five-items measuring different types of cyberbullying and cybervictimization were added, and total scores ranged from 0 to 20. Reliability coefficients at T2 were, respectively, α = 0.77 for cyberbullying and α = 0.69 for cybervictimization.

Students' involvement in school bullying and victimization was measured using the Olweus Bully/Victim Questionnaire (Olweus, 1993; Menesini et al., 1997; Baldry and Farrington, 1999). Participants were asked to rate their bullying and/or victimization experiences in the previous 6 months by answering 14 questions (seven for bullying and seven for school victimization) on a five-point scale ranging from 0 = “never” to 4 = “several times a week.” Items were then summed to create the school bullying (α = 0.60) and the school victimization (α = 0.71) scales.

Empathy was measured using the Basic Empathy Scale (Jolliffe and Farrington, 2006; Albiero et al., 2009) consisting of a total of 20 items (items for cognitive empathy and 11 items for affective empathy) measured on a five-point Likert scale ranging from 0 = “Strongly agree” to 4 = “Strongly disagree.” Reliability coefficients were, respectively, α = 0.67 for cognitive empathy and α = 0.72 for affective empathy.

Moral Disengagement was measured using the Bandura et al. (1996) scale, adapted and validated in Italian by Caprara et al. (2006), consisting of 32 items, each measured on a 5-point Likert scale ranging from 1 = “Strongly disagree” to 5 = “Strongly agree” (α = 0.91).

The Increasing Self-Awareness of Cyberbullying (ISAC) scale was developed to measure students' awareness of online risks. The scale consisting of 6 items was measured on a five-point Likert scale each ranging from 1 = “Strongly agree” to 5 = “Strongly disagree” [e.g., “Everybody could see my notice board on my social network profile(s)” and “To share online someone's photos or other materials. It is just a way to mock them”] (α = 0.74).

To measure students' perceived social support, two subscales of the Multidimensional Scale of Perceived Social Support Assessment were used (Zimet et al., 1988, 1990). Each subscale consisted of four items, each measuring perceived parental and peer support. Students rated their perception of being socially supported on a seven-point Likert scale ranging from 1 = “Strongly agree” to 7 = Strongly disagree” (respectively, α = 0.84 for parental support, α = 0.89 for peer support).

Parental online monitoring strategies, as reported by adolescents, were measured using three different items. Participants rated their parents' role in speaking with them about Internet security, giving them clear Internet use rules, and monitoring their online activities on a five-point Likert scale ranging from 1 = “Always” to 5 = “Never” (α = 0.72).

School climate was measured with a new eight-item scale (e.g., “If I have some problems, I can count on teachers' help and support” and “Most of the students support and participate with interest in all school's activities”), each measured from 1 = “Strongly Disagree” to 5 = “Strongly Agree” (α = 0.78).

### Procedure

Five schools in the Campania region, South Italy, participated in the study. Before data collection, the approval of the Department of Psychology's Ethical committee (29/2015) and the custodial adults and children's consent were obtained. Students participating in the study filled in the anonymous Tabby Improved Online Actuarial Checklist during school hours at the Computer Technology Room (CTR). Here, each student sat in front of a PC connected to the www.tabby.eu website and was told he/she had to fill in an anonymous self-report questionnaire regarding his/her experience using the new communication technologies and online experiences in the previous 6 months. The second data collection (follow-up T2) took place after 6 months, a few weeks before the end of the same school year.

Before filling in the questionnaire, the terms cyberbullying and cybervictimization were explained to have a common understanding of what was investigated. Students were assured of the confidentiality of the study and the anonymity of the answers provided. Students were allowed to pose questions. Students were also instructed about generating an ID code, allowing us to match the questionnaire anonymously with answers at T1 and those provided after 6 months (T2). The guideline provided to students was as follows: “Insert your personal code (two numbers of your date of birth- for example, 03 if you were born on the 3rd, last two letters of your surname, and the last 3 numbers of your mobile or home phone number/if you don't have it, e.g., 03BA362, for Barba born on the 3rd, with mobile nr: ++362).” After completing the questionnaire, all students returned to their classes.

### Data analyses

The data collected within the database were analyzed using the SPSS statistical package (version 21.0, IBM Milano, Milan, Italy). Descriptive statistics were carried out to assess means and standard deviations were calculated for each variable.

As preliminary analyses, simple correlations were calculated between risk factors of our predictive models to test multicollinearity. In line with Dancey and Reidy (2007), a cut-off of 0.70 indicated the absence of high correlations among predictors and the absence of multicollinearity.

Then, we used the hierarchical regression analysis to test our hypothesis using a model that considered the possible role of the individual, relational, and contextual risk factors (Bronfenbrenner, 1977, 1979) in youth onset of involvement in cyberbullying and cybervictimization behaviors. As criteria for the inclusion or exclusion of variables in each step of regression, we used a level of *F* < 0.05. We assessed statistical significance at least at a 0.05 level for all statistical analyses performed. We performed separate analyses for onset risk factors for cyberbullying and cybervictimization involvement measured at baseline (T1), by excluding from the following analyses all students who at baseline (T1) declared to be involved in cyberbullying and cybervictimization and including only students that at follow-up (T2) were involved in cyberbullying and cybervictimization.

## Results

### Preliminary analyses of onset risk factors for cyberbullying involvement

A total of 286 students (40.2% male students) aged between 10 and 16 years (*M* = 12.16, *SD* = 1.34) were included in the following analyses aimed at investigating onset risk factors for cyberbullying involvement. Descriptive statistics for onset risk factors measured at T1 were calculated (see Table 1).

**Table 1:** Descriptive statistics of onset cyberbullying (*N* = 286).

	***M***	**SD**	**Min**	**Max**
School bullying	0.60	1.37	0	14
School victimization	2.24	3.37	0	18
Low awareness online risk	5.58	4.79	0	24
Low cognitive empathy	9.69	4.67	0	29
Low affective empathy	14.67	6.81	0	34
High moral disengagement	66.95	19.89	32	141
Low parental support	7.10	4.11	4	28
Low parental online activities monitoring	6.02	3.03	0	12
Low peers support	9.34	5.25	4	28
Poor school climate	7.67	5.13	0	27

As shown in Table 2, the maximum observed coefficient of 0.50 between affective empathy and cognitive empathy is a value below the cut-off of 0.70. Looking in more detail at the correlation matrix, following Cohen's interpretation of *r*-values (high correlation for *r* > 0.40 and moderate correlation for 0.40 < *r* < 0.20, 1,988), we observed a high correlation between moral disengagement and awareness of online risks (*r* = 0.40, *p* < 0.001), the two dimensions of empathy, i.e., affective and cognitive (*r* = 0.50, *p* < 0.001), the two kinds of support, i.e., support of friends and support of parents (*r* = 0.44, *p* < 0.001), and between support of friends and school climate (*r* = 0.45, *p* < 0.001). This last high correlation was not surprising given that many adolescents developed friendships in the school context. School bullying and school victimization were highly associated with each other (*r* = 0.40, *p* < 0.001).

**Table 2:** Correlation matrix for onset of cyberbullying.

	**1**	**2**	**3**	**4**	**5**	**6**	**7**	**8**	**9**	**10**
1. Moral disengagement	–	0.02	0.18^**^	−0.08	0.06	0.40^***^	0.14^*^	0.25^***^	0.20^***^	0.19^***^
2. School victimization		–	0.40^***^	−0.04	0.09	0.01	0.31^***^	0.02	−0.06	0.19^**^
3. School bullying			–	−0.11	−0.01	0.02	0.12^*^	0.14^*^	−0.02	0.14^*^
4. Low cognitive empathy				–	0.50^***^	−0.01	0.11^*^	−0.01	0.22^***^	0.29^***^
5. Low affective empathy					–	−0.01	0.04	−0.06	0.25^***^	0.21^***^
6. Low awareness online risks						–	0.12^*^	0.17^**^	0.29^***^	0.01
7. Low peer support							–	0.44^***^	0.23^***^	0.45^***^
8. Low parents support								–	0.23^***^	0.34^***^
9. Low parental online monitoring									–	0.26^***^
10. Poor school climate										**–**

^*^p < 0.05,

^**^p < 0.01,

^***^p < 0.001.

Further, a moderate correlation emerged between moral disengagement and support of parents (*r* = 0.25, *p* < 0.001), and school victimization and support of friends (*r* = 0.31, *p* < 0.001). Both cognitive and affective empathy showed moderate correlation with parental online activities monitoring (*r*_cognitiveempathy_ = 0.22, *p* < 0.001; *r*_affectiveempathy_ = 0.25, *p* < 0.001) and school climate (*r*_cognitiveempathy_ = 0.29, *p* < 0.001; *r*_affectiveempathy_ = 0.21, *p* < 0.001). Similar to empathy, the support of parents showed a moderate correlation with both parental online activities monitoring (*r* = 0.23, *p* < 0.001) and school climate (*r* = 0.34, *p* < 0.001). Parental control also resulted in a moderate correlation with support of friends (*r* = 0.23, *p* < 0.001) and with school climate (r= 0.26, *p* < 0.001).

### Regression analyses: Onset risk factors for cyberbullying involvement

The stepwise regression for bullying and victimization predicted four significant steps (Table 3). In the first step, only awareness of online risks was statistically significant for cyberbullying behaviors. Low awareness of online risks predicted the involvement in cyberbullying behavior after 6 months: β = 0.28, *t*_(1, 276)_ = 4.89, *p* < 0.001, 95% C.I. = 0.06, 0.14. In the first step, the regression model explained 8.0% of the total variance, *F*_(1, 276)_ = 23.98, *p* < 0.001.

**Table 3:** Multiple linear regression analysis (stepwise) results regarding onset of cyberbullying.

**Variable**	**B**	**SE B**	**β**	***t***	***R^2^***	**Δ*R^2^***
Step 1					0.80	0.77
Constant	−0.17	0.15		−1.01		
Low awareness online risks	0.10	0.02	0.28	4.90^***^		
Step 2					0.13	0.12
Constant	−0.33	0.16		−2.08^*^		
Low awareness online risks	0.10	0.02	0.28	4.94^***^		
School bullying	0.27	0.07	0.22	3.82^***^		
Step 3					0.15	0.14
Constant	−0.49	0.17		−2.93^**^		
Low awareness online risks	0.09	0.02	0.27	4.79^***^		
School bullying	0.25	0.07	0.20	3.51^***^		
Gender (male = 1)	0.51	0.20	0.14	2.53^*^		
Step 4					0.17	0.15
Constant	0.64	0.47		1.36		
Low awareness online risks	0.09	0.02	0.27	4.82^***^		
School bullying	0.24	0.07	0.19	3.43^***^		
Gender (male = 1)	0.65	0.21	0.18	3.13^**^		
Low affective empathy	−0.05	0.02	−0.15	−2.58^**^		

^*^p < 0.05,

^**^p < 0.01,

^***^p < 0.001.

In the second step, school bullying became a significant predictor of cyberbullying behaviors. A higher level of school bullying predicted involvement in cyberbullying behavior: β = 0.22, *t*_(2, 275)_ = 3.82, *p* < 0.001, 95% C.I. = 0.13, 0.41, and awareness of online risks was still a significant predictor in step 2 of the regression model, β = 0.28, *t*_(2, 275)_ = 4.94, *p* < 0.001, 95% C.I. = 0.06, 0.14. In the second step, the regression model explained 13.0% of the total variance with an increased value of 5.0%, *F*_change(1, 275)_ = 14.61, *p* < 0.001.

In the third step, gender became a significant predictor of cyberbullying behaviors. Being male predicted higher cyberbullying behaviors β = 0.14, *t*_(3, 274)_ = 2.52, *p* = 0.012, 95% C.I. = 0.11, 0.91. Awareness of online risks was still a significant predictor in step 3 of the regression model, β = 0.27, *t*_(3, 274)_ = 4.79, *p* < 0.001, 95% C.I. = 0.06, 0.14, as well as school bullying β = 0.20, *t*_(3, 274)_ = 3.51, *p* = 0.001, 95% C.I. = 0.11, 0.39. In the third step, the regression model explained 15.0% of the total variance with an increased value of 2.0%, *F*_change(1, 274)_ = 6.37, *p* = 0.012.

In the fourth and final step, affective empathy emerged as a significant predictor of cyberbullying behaviors, a higher level of affective empathy predicted a high level of cyberbullying behavior, β = −0.15, *t*_(4, 273)_ = −2.58, *p* < 0.001, 95% C.I. = 0.06, 0.14. Predictors that were significant at previous steps, were still significant at the final steps, i.e., awareness of online risks, β = 0.27, *t*_(4, 273)_ = 4.81, *p* < 0.001, 95% C.I. = 0.06, 0.14; school bullying act β = 0.19, *t*_(4, 273)_ = 3.43, *p* = 0.001, 95% C.I. = 0.10, 0.38; gender β = 0.18, *t*_(4, 273)_ = 3.13, *p* = 0.002, 95% C.I. = 0.24, 1.05. The final step explained 17.0% of the total variance, with an increased value of 2.0% compared with the third step, *F*_change(1, 273)_ = 6.67, *p* = 0.01.

### Prelaminar analyses of onset risk factors for cybervictimization involvement

About 175 students (48.6% males), aged between 10 and 16 years (*M* = 12.23, *SD* = 1.36), were included in the following analyses aimed at investigating onset risk factors for cybervictimization involvement. Descriptive statistics for onset risk factors measured at T1 were calculated (see Table 4).

**Table 4:** Descriptive statistics of onset of cybervictimization (*N* = 175).

	**M**	**SD**	**Min**	**Max**
School bullying	0.61	1.33	0	10
School victimization	1.40	2.72	0	18
Low awareness online risk	5.64	4.92	0	24
Low cognitive empathy	16.49	4.28	3	33
Low affective empathy	25.35	6.10	2	39
High moral disengagement	7.05	3.99	4	28
Low parental support	3.88	2.30	0	9
Low parental online activities monitoring	8.48	4.58	4	28
Low peers support	7.20	4.91	0	20
Poor school climate	0.61	1.33	0	10

Moving to the cybervictims behaviors, as shown in Table 5, the maximum observed coefficient of 0.565 between support of friends and support of parents was below the cut-off of 0.70 indicating the absence of high correlations among predictors and the absence of multicollinearity (Dancey and Reidy, 2007). Reviewing the correlation matrix in more detail following Cohen's interpretation of *r*-values (1988), a high correlation emerged between cognitive and affective empathy (*r* = 0.32, *p* < 0.001). Further, moral disengagement was highly correlated with school bullying (*r* = 0.32, *p* < 0.001), awareness of online risks (*r* = 0.43, *p* < 0.001), parental monitoring of online activities (*r* = 0.31, *p* < 0.001), and school climate (*r* = 0.32, *p* < 0.001). School climate showed a high correlation with school bullying (*r* = 0.34, *p* < 0.001), and both support of parents (*r* = 0.40, *p* < 0.001) and peers (*r* = 0.40, *p* < 0.001), by confirming the importance of the school context. Further, a moderate correlation emerged between moral disengagement and support of parents (*r* = 0.27, *p* < 0.001) as well as school victimization and peer support (*r* = 0.31, *p* < 0.001). School bullying resulted in a moderate correlation with support of parents (*r* = 0.26, *p* < 0.001), parental monitoring of online activities (*r* = 0.26, *p* < 0.001), and school climate (*r* = 0.34, p < .001). Both cognitive and affective empathy showed a moderate correlation with school climate (*r*_cognitiveempathy_ = 0.26, *p* < 0.001; *r*_affectiveempathy_ = 0.23, *p* < 0.001). Cognitive empathy had a moderate correlation with peer support (*r* = 0.21, *p* = 0.002), whereas affective empathy showed a moderate correlation with parental monitoring of online activities (*r* = 0.28, *p* < 0.001). Awareness of online risks resulted in a moderate correlation with peer support (*r* = 0.20, *p* = 0.008), parental support (*r* = 0.23, *p* = 0.003), and parental monitoring of online activities (*r* = 0.27, *p* < 0.001). Finally, both parental support (*r* = 0.27, *p* < 0.001) and peer support (*r* = 0.25, *p* < 0.001) were moderately correlated to parental monitoring of online activities.

**Table 5:** Correlation matrix for onset of cybervictimization.

	**1**	**2**	**3**	**4**	**5**	**6**	**7**	**8**	**9**	**10**
1. Moral disengagement	–	−0.01	0.32^***^	−0.12	0.09	0.43^***^	0.01	0.27^***^	0.31^***^	0.32^***^
2. School victimization		–	0.12	0.08	0.14	0.10	0.20^**^	0.05	0.18^*^	0.11
3. School bullying			–	0.07	0.13	0.12	0.05	0.26^***^	0.26^***^	0.34^***^
4. Low cognitive empathy				–	0.54^***^	−0.11	0.21^**^	−0.03	0.18^*^	0.26^***^
5. Low affective empathy					–	−0.04	0.03	−0.05	0.28^***^	0.23^**^
6. Low awareness online risks						–	0.20^**^	0.23^**^	0.27^***^	0.18^*^
7. Low peer support							–	0.57^***^	0.25^***^	0.40^***^
8. Low parents support								–	0.27^***^	0.40^***^
9. Low parental online monitoring									–	0.27^***^
10. Poor school climate										–

^*^p < 0.05,

^**^p < 0.01,

^***^p < 0.001.

### Regression analyses: Onset of cybervictimization involvement

The stepwise regression model for bullying and victimization predicted five significant steps (Table 6). In the first step, only affective empathy was a statistically significant predictor of cybervictimization behaviors. A high level of affective empathy predicted a high level of cybervictimization β = −0.29, *t*_(1, 167)_ = −3.86, *p* < 0.001, 95% C.I. = −0.14, −0.05. In the first step, the regression model explained 8.0% of the total variance, *F*_(1, 167)_ = 14.90, *p* < 0.001.

**Table 6:** Multiple linear regression analysis (stepwise) results regarding onset of cybervictimization.

**Variable**	**B**	**SE B**	**β**	***t***	***R^2^***	**Δ*R^2^***
**Step 1**					**0.08**	**0.08**
Constant	3.22	0.65		4.99^***^		
Low affective empathy	−0.09	0.03	−0.29	−3.86^***^		
Step 2					0.16	0.15
Constant	3.31	0.62		5.34^***^		
Low affective empathy	−0.11	0.02	−0.33	−4.54^***^		
School bullying	0.41	0.11	0.28	3.91^***^		
Step 3					0.19	0.17
Constant	2.31	0.74		3.10^**^		
Low affective empathy	−0.11	0.02	−0.33	−4.70^***^		
School bullying	0.33	0.11	0.23	3.02^**^		
High moral disengagement	0.02	0.01	0.18	2.37^*^		
Step 4					0.21	0.19
Constant	2.52	0.74		3.38^***^		
Low affective empathy	−0.12	0.02	−0.37	−5.09^***^		
School bullying	0.30	0.11	0.20	2.71^**^		
High moral disengagement	0.01	0.01	0.15	2.00^*^		
Gender (male = 1)	0.59	0.30	0.15	1.99^*^		
Step 5					0.23	0.20
Constant	2.52	0.74		3.41^***^		
Low affective empathy	−0.13	0.02	−0.39	−5.38^***^		
School bullying	0.27	0.11	0.19	2.49^*^		
High moral disengagement	0.02	0.01	0.16	2.11^*^		
Gender (male = 1)	0.61	0.29	0.16	2.07^*^		
School victimization	0.10	0.05	0.14	2.02^*^		

^*^p < 0.05,

^**^p < 0.01,

^***^p < 0.001.

In the second step, school bullying became a significant predictor of cybervictimization. A higher level of involvement in school bullying predicted a higher level of cybervictimization β = 0.28, *t*_(2, 166)_ = 3.90, *p* < 0.001, 95% C.I. = 0.20, 0.61. Affective empathy was still a significant predictor in step 2 of the regression model, β = −0.33, *t*_(2, 166)_ = −4.70, *p* < 0.001, 95% C.I. = −0.16, −0.06. In the second step, the regression model explained 16.0% of the total variance, with an increased value of 8.0%, *F*_change(1, 166)_ = 15.25, *p* < 0.001.

In the third step, moral disengagement became a significant predictor of cybervictimization. A higher level of moral disengagement predicted involvement in cybervictimization after 6 months β = 0.18, *t*_(3, 165)_ = 2.34, *p* = 0.019, 95% C.I. = 0.01, 0.03. Affective empathy was still a significant predictor in step 3 of the regression model, β = −0.33, *t*_(3, 165)_ = −4.70, *p* < 0.001, 95% C.I. = −0.16, −0.06, as well as school bullying β = 0.22, *t*_(3, 165)_ = 3.02, *p* = 0.003, 95% C.I. = 0.11, 0.54. In the third step, the regression model explained 19.0% of the total variance, with an increased value of 3.0%, *F*_change(1, 165)_ = 3.98, *p* = 0.019.

In the fourth step, gender emerged as a significant predictor of cybervictimization. Being male predicted a higher level of cybervictimization β = 0.15, *t*_(4, 164)_ = 1.99, *p* = 0.048, 95% C.I. = 0.01, 1.17. Affective empathy, β = −0.37, *t*_(4, 164)_ = −5.09, *p* < 0.001, 95% C.I. = −0.17, −0.08; school bullying, β = 0.20, *t*_(4, 164)_ = 2.70, *p* = 0.008, 95% C.I. = 0.08, 0.51, and moral disengagement, β = 0.15, *t*_(4, 164)_ = 2.01, *p* = 0.047, 95% C.I. = 0.01, 0.03, were still significant in the fourth step of the regression model. The fourth step explained 21.0% of the total variance, with a further increased value of 2.0% compared with the third step, *F*_change(1, 164)_ = 3.98, *p* = 0.048.

In the fifth and final step of the regression model, school victimization emerged as a significant predictor of cybervictimization, a higher level of school victimization predicted a higher level of cybervictimization behaviors, β = 0.154, *t*_(5, 163)_ = 2.02, *p* = 0.045, 95% C.I. = 0.01, 0.20. Crucially, all predictors that were significant in previous steps of the regression model were still significant in the final step of the regression: Affective empathy, β = −0.39, *t*_(5, 163)_ = −5.38, *p* < 0.001, 95% C.I. = −0.18, −0.08; school bullying, β = 0.19, *t*_(5, 164)_ = 2.48, *p* = 0.014, 95% C.I. = 0.06, 0.49; moral disengagement, β = 0.16, *t*_(5, 163)_ = 2.12, *p* = 0.036, 95% C.I. = 0.01, 0.03; and gender, β = 0.16, *t*_(5, 163)_ = 2.08, *p* = 0.040, 95% C.I. = 0.03, 1.18. The final step of the regression model explained 23.0% of the total variance, with an increased value of 2.0% compared with the previous step, *F*_change(1, 163)_ = 4.06, *p* = 0.045.

## Discussion

As far as we know, to date, no longitudinal studies on risk factors for cyberbullying and cybervictimization have been carried out adopting the criminological concept of “onset”. The current study aimed to investigate the onset risk factors for youth involvement in cyberbullying and cybervictimization by conducting a short-term longitudinal study involving 286 Italian students aged between 10 and 16 years. To this aim, onset risk factors for both cyberbullying and cybervictimization involvement were analyzed separately and by excluding from our analyses all students that at baseline (T1) declared to be involved in cyberbullying or in cybervictimization.

Concerning participants' onset risk factors for cyberbullying, our results highlighted that awareness of online risks, involvement in school bullying, and gender were all significantly associated with youth involvement in cyberbullying after 6 months. Specifically, consistent with previous research, our findings indicate that onset of cyberbullying in youth is predicted by low levels of awareness of online risks (Camerini et al., 2020), previous involvement in school bullying (Kowalski et al., 2014, 2019; Baldry et al., 2015; Guo, 2016; Chen et al., 2017; Cosma et al., 2020; Estévez et al., 2020), and being male (Barlett et al., 2021; Giordano et al., 2021).

Surprisingly, high levels of affective empathy were found to be significant onset risk factors for involvement in cyberbullying after 6 months. Although this finding was unexpected at first glance, as underlined by a recent systematic review and meta-analysis, cyberbullies scored lower in cognitive and affective empathy (Zych et al., 2019b). However, based on the more general literature about aggressive behaviors (Vachon et al., 2014) we can hypothesize the existence of more than two components of empathy, as, for instance, cognitive empathy, affective resonance, and affective dissonance (Vachon and Lynam, 2016). As the affective dissonance dimension is associated with aggressive and externalizing behaviors (Vachon and Lynam, 2016), it could be possible that in our study, high affective empathy predicted cyberbullying involvement, as those students reported higher capability to access victims' emotions to use them to take pleasure in others' pain. Future studies are needed to investigate the possible role of these three dimensions of empathy in youth onset involvement in cyberbullying.

Contrary to our expectations, no significant associations were found between low levels of cognitive empathy, low levels of perceived parental online monitoring and support, low levels of peer support and negative school climate, and the onset of cyberbullying behaviors.

On the onset risk factors for cybervictimization, our results highlighted that affective empathy, involvement in school bullying and victimization, and gender were all significantly associated with youth involvement in cybervictimization after 6 months. Specifically, our findings indicate that onset of cybervictimization in youth is predicted by high levels of affective empathy, previous involvement in school bullying and school victimization, and being male.

Concerning the relationship between gender and cybervictimization, even if contrasting results were reported in the literature, our findings are consistent with those reported by Huang et al. (2019) and Rao et al. (2019); boys were more at risk than girls of being cybervictims.

Consistent with our results, involvement in school victimization, as evidenced in many previous studies, is a significant predictor of cybervictimization (Kowalski et al., 2014; Baldry et al., 2015; Estévez et al., 2020; Oriol et al., 2021; Rodríguez-Álvarez et al., 2021).

However, our results also support the “role inversion hypothesis” (Ybarra and Mitchell, 2004; Cuadrado-Gordillo and Fernández-Antelo, 2014, 2019; Baldry et al., 2016), which is the possibility of being cybervictimized as an act of revenge for being a school bully, confirming that independently of the role held in peer aggressive behaviors, school bullying and victimization are crucial risk factors for youth involvement in cybervictimization after 6 months.

Moreover, high levels of affective empathy were found to significantly affect participants' involvement in cybervictimization after 6 months, confirming the results of a recent meta-analysis (Zych et al., 2019b) that found that cybervictims reported high levels of affective empathy than non-cybervictims.

Furthermore, we also found that high levels of moral disengagement measured at baseline predicted the involvement in cybervictimization at follow-up, consistent with Pornari and Wood (2010), Kowalski et al. (2014), Chen et al. (2017), and Parlangeli et al. (2020), hypothesizing that youth with the tendency of blaming the victims and justifying violent behaviors were probably less aware of their risk of being cybervictimized.

Contrary to our hypotheses, the onset of cybervictimization was not significantly associated with being female, reporting low levels of online risk awareness, high cognitive empathy, feeling not supported by parents and monitored about their online activities, perceiving low levels of support by peers, and a negative school climate.

## Practical implications

The results underline the existence of a different pattern of onset risk factors for cyberbullying and cybervictimization, confirming the role of some of the more investigated risk factors for cyberbullying and cybervictimization, such as school bullying and victimization and gender.

However, even if our results are consistent with previous research on risk factors for cyberbullying and cybervictimization (Kowalski et al., 2014; Baldry et al., 2015; Zych et al., 2019a; Camerini et al., 2020), at the same time, they underline the existence of different patterns for youth onset involvement in cyberbullying and cybervictimization suggesting several implications for the development of further prevention and intervention programs.

Specifically, according to our results, it seems necessary to work on the implementation of holistic anti-cyberbullying programs which can adapt the nature and the type of intervention differentiating between prevention and sensitization activities from those aimed at targeting cyberbullies and cybervictims.

Prevention and sensitization programs should include specific curricula for identifying, assessing, and managing the possible “alarm bells” associated with the onset of peer aggressive behaviors such as cyberbullying and cybervictimization; this is to intervene before adolescents' involvement in such behaviors, differentiating between individual, relational, and contextual risk factors associated with the involvement as perpetrator and victim. For instance, according to our results, it could be useful for preventing youth involvement in cyberbullying to consider and implement specific modules on children and youth socioemotional abilities, focusing on empowering the affective resonance dimension while managing the affective dissonance one. Though in terms of prevention, for cybervictims, activities should focus on investigating youth's previous involvement in school bullying dynamics, and in particular, understand the possible role overlap or inversion between the involvement as school bullies or victims and the subsequent experience of cybervictimization.

The development of such prevention and intervention programs based on individual, relational, and contextual onset risk factors for cyberbullying and cybervictimization should overcome one of the main limits of current anti-bullying programs, which is their limited efficacy in preventing and reducing such behaviors over time (Polanin et al., 2021; Lan et al., 2022).

## Limitations and future research

This study has some limitations that should be addressed in future studies. First, as common in longitudinal studies, we observed a mortality ratio of 27.0% of the total sample (*N* = 122) at T2, due mainly to the one participating school dropout. Despite this limitation, the longitudinal design of our study allowed us to evaluate the causal relationship between the onset risk factors for cyberbullying and cybervictimization and the youth's involvement in such behaviors after 6 months. Furthermore, we also performed attrition rate analyses to check that the retaining sample was representative of the initial one.

Another possible limitation of our study is related to sample size, thus affecting the generalizability of our results. The small sample size involved in our analyses arises from the need to analyze how individual, relational, and contextual risk factors measured at baseline influence our participants' consequent involvement (after 6 months) in cyberbullying and cybervictimization.

Even if the reliability coefficient of some scales at T1 were around 0.60, these values should be considered acceptable given the short scales dimension (Gliem and Gliem, 2003). Future cross-cultural studies should help to verify the scales' reliability across different countries. Another limitation of the present research is the low percentage of variance explained by our hierarchical regression models that were tested (~20.0%). This low power can be framed by considering the cyberbullying and cybervictimization nature, as all social complex phenomena, the involvement in such behaviors could be affected by the interaction of several individual, relational, and contextual factors. Despite this limitation, the identification of a different pattern of onset risk factors influencing youth involvement in cyberbullying and cybervictimization could represent a turning point for the development of effective primary prevention and promotion strategies.

Future research is needed to investigate the onset risk factors for involvement in cyberbullying and cybervictimization by implementing long-term longitudinal studies to assess their trajectories and patterns over time.

Second, another possible limitation is that our measures were self-reported, maybe eliciting participants' social desirability or leading them to underestimate their involvement in cyberbullying and cybervictimization.

## Conclusion

We investigated the onset of individual, relational, and contextual risk factors for youth and adolescents' involvement in cyberbullying and cybervictimization by involving a sample of Italian students in a short-term longitudinal study (a follow-up after 6 months). Overall, we found the existence of a different pattern of risk factors influencing adolescents' onset of cyberbullying and cybervictimization. Specifically, our results showed that being male, involvement in school bullying, low levels of awareness of online risk, and high levels of affective empathy were all significant onset risk factors for cyberbullying. Being male, involvement in school bullying and victimization, high levels of affective empathy, and moral disengagement were found to be onset risk factors for cybervictimization.

These results, underline the need to develop and implement holistic anti-cyberbullying programs that can adapt to the nature and the type of intervention, differentiating between prevention and sensitization activities from those aimed at targeting cyberbullies and cybervictims. Programs should include specific curricula for identifying, assessing, and managing the possible “alarm bells” associated with the onset of cyberbullying and cybervictimization; this is to intervene before adolescents' involvement in such behaviors, differentiating between individual, relational, and contextual risk factors associated with the involvement as perpetrator and victim.

## Data availability statement

The raw data supporting the conclusions of this article will be made available by the authors, without undue reservation.

## Ethics statement

The studies involving human participants were reviewed and approved by Department of Psychology, University of Campania Luigi Vanvitelli. Written informed consent to participate in this study was provided by the participants' legal guardian/next of kin.

## Author contributions

AS designed the work. AA and AS analyzed the data results and revised the manuscript. AS, AA, AE, MS, and DA drafted the manuscript. All authors contributed to the article and approved the submitted version.
